# *Ganoderma lucidum* Spore Polysaccharide Inhibits the Growth of Hepatocellular Carcinoma Cells by Altering Macrophage Polarity and Induction of Apoptosis

**DOI:** 10.1155/2021/6696606

**Published:** 2021-03-05

**Authors:** Ming Song, Zhen-hao Li, Hong-shun Gu, Ru-ying Tang, Rui Zhang, Ying-li Zhu, Jin-lian Liu, Jian-jun Zhang, Lin-yuan Wang

**Affiliations:** ^1^School of Traditional Chinese Medicine, Beijing University of Chinese Medicine, Beijing 100029, China; ^2^Zhejiang Shouxiangu Institute of Rare Medicine Plant, Wuyi, 321200, China; ^3^Beijing Cairui Medicine Technology Institute, Beijing 100094, China; ^4^School of Chinese Materia Medica, Beijing University of Chinese Medicine, Beijing 100029, China

## Abstract

**Background:**

*Ganoderma lucidum* has certain components with known pharmacological effects, including strengthening immunity and anti-inflammatory activity. *G. lucidum* seeds inherit all its biological characteristics. *G. lucidum* spore polysaccharide (GLSP) is the main active ingredient to enhance these effects. However, its specific biological mechanisms are not exact. Our research is aimed at revealing the specific biological mechanism of GLSP to enhance immunity and inhibit the growth of H22 hepatocellular carcinoma cells.

**Methods:**

We extracted primary macrophages (M*ø*) from BALB/c mice and treated them with GLSP (800 *μ*g/mL, 400 *μ*g/mL, and 200 *μ*g/mL) to observe its effects on macrophage polarization and cytokine secretion. We used GLSP and GLSP-intervened macrophage supernatant to treat H22 tumor cells and observed their effects using MTT and flow cytometry. Moreover, real-time fluorescent quantitative PCR and western blotting were used to observe the effect of GLSP-intervened macrophage supernatant on the PI3K/AKT and mitochondrial apoptosis pathways.

**Results:**

In this study, GLSP promoted the polarization of primary macrophages to M1 type and the upregulation of some cytokines such as TNF-*α*, IL-1*β*, IL-6, and TGF-*β*1. The MTT assay revealed that GLSP+M*ø* at 400 *μ*g/mL and 800 *μ*g/mL significantly inhibited H22 cell proliferation in a dose-dependent manner. Flow cytometry analysis revealed that GLSP+Mø induced apoptosis and cell cycle arrest at the G2/M phase, associated with the expression of critical genes and proteins (PI3K, p-AKT, BCL-2, BAX, and caspase-9) that regulate the PI3K/AKT pathway and apoptosis. GLSP reshapes the tumor microenvironment by activating macrophages, promotes the polarization of primary macrophages to M1 type, and promotes the secretion of various inflammatory factors and cytokines.

**Conclusion:**

Therefore, as a natural nutrient, GLSP is a potential agent in hepatocellular carcinoma cell treatment and induction of apoptosis.

## 1. Introduction

Hepatocellular carcinoma (HCC) is the most life-threatening disease worldwide, having high mortality and poor prognosis and an incidence of more than one million cases per year [1]. At present, the treatments for liver cancer are surgery, radiotherapy, and chemotherapy [2]. Its occurrence and development are closely related to various molecular mechanisms in the cell. Recently, an increasing number of chemical drugs and new targeted drugs have been developed. However, some patients are still resistant to drugs. Therefore, the development of new natural medicines is expected to become another strategy for treating liver cancer. Active extracts of various natural medicinal plants have been tested for cancer treatment and have shown good antitumor efficacy [3].

Nowadays, researches have focused on the immunomodulation and antitumor activity of natural products, and this has become the focus of emerging research [4]. Naturally sourced antitumor drugs have been shown to exhibit therapeutic effects and few adverse reactions in tumor therapy. They can repair the body's immune system and even cure tumors [5]. *Ganoderma lucidum*, which is also called “Lingzhi” has been used medicinally for more than 2000 years [6] and has been regarded as an effective medicinal compound, reinforcing healthy qi to restore normal function and prolong life and has almost no toxic side effects [7]. *G. lucidum* spores are microscopic and are ejected from the cap during growth and maturation. These germ cells have all of *G. lucidum* genetically active substances [8]. Modern pharmacological studies have shown that *G. lucidum* spores have antitumor effects, increase immune regulation, lower blood sugar and lipid, increase anti-inflammatory and antihypoxia ability, and scavenge free radicals [9].

Macrophages (Mø) play an essential role in humoral and cellular immunity and in maintaining tissue homeostasis [10]. Related studies have found that macrophages are incredibly plastic and can be activated into a series of continuously adjustable functional states under the stimulation of different environments or drugs [11]. Classically activated (M1 type) macrophages and alternatively activated (M2 type) macrophages are the two extremes of this state. The process by which naive (M*φ* type) macrophages are stimulated by exogenous factors in specific tissues to differentiate into M1 or M2 macrophages is called macrophage polarization [12]. The dynamic balance between M1 and M2 is vital for maintaining homeostasis. Once the balance is broken, the human body faces a variety of diseases that can sometimes be treated with drugs to regulate these macrophages. The transformation of M1 and M2 macrophages is a dynamic and reversible process. Directional polarization may provide new methods for cancer treatment [13, 14].

Tumor-associated macrophages (TAMs) are similar to the function of immune cells in the tumor microenvironment and mainly infiltrate the tumor matrix to mediate inflammation [15]. The secretion of cytokines, chemokines, growth factors, and proteases and the regulation of intracellular signaling pathways play a vital role in modulating the function of TAMs and tumor cells. The tumor microenvironment combines chronic inflammation, low oxygen levels, nutritional deficiencies, and acidosis, creating extremely complex dynamic systems [16] that regulate tumor growth, proliferation, metastasis, and immune escape. Therefore, we think that the treatment of tumors by reducing the stress state of the tumor's internal environment and then feeding it back to the tumor cells may promote tumor cell apoptosis or autophagy [17, 18]. Supernatant transfer of various cell cocultures in vitro has been used to mimic the tumor microenvironment [19].

By comparing the content and composition of GLSP and *G. lucidum* polysaccharides (GLP), we found that the overall structure is similar, but there are still many differences. At present, more than 200 kinds of substances have been separated, of which the largest is *β*-glucan and a few are *α*-glucan [20]. Although there have been many studies on GLP, because the shell of *G. lucidum* spores is hard and difficult to remove completely, we apply a brand-new removal wall technology that makes it possible to extract GLSP with higher purity. GLSP has better physical and chemical properties than GLP, and its application prospects are broader [21, 22]. Besides, GLSP plays a vital role in nourishing and protecting the liver, resisting radiation, resisting gene mutations, and resisting inflammation. Such effects have not been confirmed in GLP-related studies.

**Figure 1:**
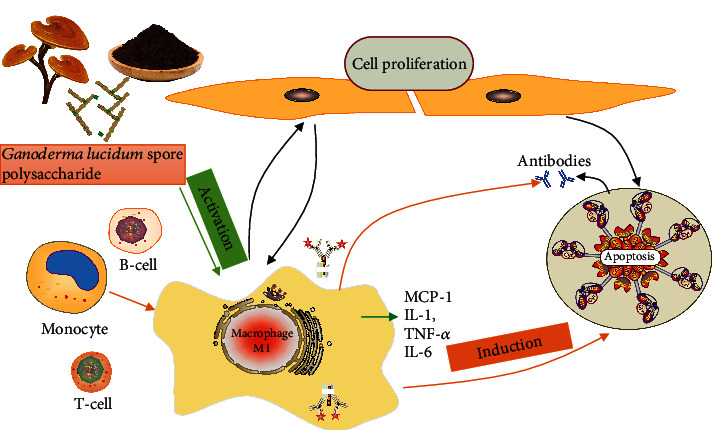
GLSP enhances immunity and induces tumor cell apoptosis by activating macrophages. GLSP: *Ganoderma lucidum* spore polysaccharide.

In our previous experiments, we found that the *G. lucidum* spore water extract had no inhibitory effect on H22 liver cancer cells and no cytotoxicity. However, when added to macrophages, it had a significant inhibitory effect on H22 liver tumor cells. To clarify the antitumor mechanism of *G. lucidum* spores, we studied the antitumor activity of *G. lucidum* spore polysaccharides (GLSP). We speculate that it is one of the targets of liver cancer, as shown in Figure 1.

## 2. Materials and Methods

### 2.1. Cells and Animals

Mouse H22 cells were obtained from Jiangsu KeyGEN BioTECH Co., Ltd. The culture conditions were 90% RMPI1640 medium + 10% FBS, cultured in an incubator at 37°C, 5% CO_2_, and saturated humidity. Sixteen BALB/c mice (male; age range, 4-6 weeks), weighing 20.0 ± 2.0 g, were obtained from the Vital River Laboratory Animal Technology Limited Company (Beijing, China). The laboratory condition is at room temperature (25 ± 2°C) and humidity (65 ± 5%). The ethics committee of Beijing University of Chinese Medicine and the China Academy of Chinese Medicine Sciences approved all the experiments (No. 2016-0012).

### 2.2. Primary Macrophage Extraction

Mice about six weeks old were shaved on a clean bench for disinfection. The animals were euthanized by cervical dislocation, and they were then immersed in 75% alcohol for 3-5 s by the tail. The mice were then fixed on the dissection table. After scrubbing the peritoneal wall with 75% alcohol, 1 mL precooled PBS was injected into the abdominal cavity with a 5 mL syringe, and the abdomen was gently massaged for 2-3 min. Under aseptic conditions, the abdominal wall was opened, the peritoneum was exposed, and the abdominal wall was scrubbed with 75% alcohol. The peritoneal fluid was aspirated with a syringe and centrifuged at 4°C at 1000 rpm/min for 10 min. Finally, 10% calf serum RPMI-1640 solution was used to suspended cells. Peritoneal macrophages were collected, viable cells > 95% with trypan blue staining were collected, the cell concentration was adjusted to 5.0 × 10^5^ cells/mL with RPMI-1640 medium, and they were inoculated into culture flasks and placed in a 5% CO_2_, 37°C incubator. After 4 hours of culture, the nonadherent cells were washed with PBS to obtain purified peritoneal macrophages.

### 2.3. Preparation of *Ganoderma lucidum* Spore Powder

**Figure 2:**
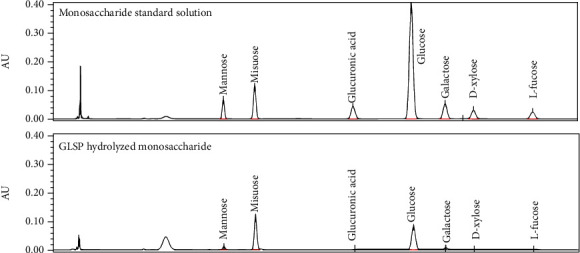
HPLC chromatograms of standard monosaccharide solution and GLSP hydrolysate.

A total of 80 g of the wall-removed G. lucidum spore powder was extracted with 95% ethanol in a 5000 mL round flask. After removing ethanol, the residue was added 2400 mL of water (30 times the amount of water) and refluxed for 3 h. The solution was then filtered and concentrated to 80 mL. Then, 425 mL of 95% ethanol diluted to 80% ethanol was added while stirring, let stand for 12 h at 0-4°C, and filtered. The precipitate was taken and dissolved in water and chloroform with n-butanol (5 : 1) mixed solution for extraction according to the Sevag method. Then, the solution was shaken for 15 min, the organic layer was removed, and the extraction was repeated four times, concentrated, and dried to obtain GLSP. The GLSP content was 92.7% according to the test method of the 2015 edition of the “Pharmaceuticals of the People's Republic of China.” HPLC chromatograms of standard monosaccharide solution and GLSP hydrolysate are shown in Figure 2.

### 2.4. Modeling and Drug Delivery

H22 cells were treated for 24 h under different conditions: only DMEM (control), GLSP (800 *μ*g/mL, 400 *μ*g/mL, and 200 *μ*g/mL), macrophage supernatant, and GLSP (800 *μ*g/mL, 400 *μ*g/mL, and 200 *μ*g/mL)+macrophage supernatant combination. The concentrations of GLSP (800 *μ*g/mL, 400 *μ*g/mL, and 200 *μ*g/mL) used in this study resulted in no inhibitory activity on macrophage growth. Culture supernatants were collected to measure levels of TNF-*α*, TGF-*β*1, IL-6, and IL-1*β*. Each group of cells was subsequently harvested to determine intracellular reactive oxygen species (ROS) production for western blot (WB) analyses and other experiments.

### 2.5. MTT Assay

H22 cells were digested, counted, and prepared into a cell suspension at a concentration of 5 × 10^4^ cells/mL. The plate was placed at 37°C, 5% CO_2_ for 24 h in a box. The drug was diluted with complete medium to the required concentration (200 *μ*g/mL, 400 *μ*g/mL, and 800 *μ*g/mL). Then, 100 *μ*L of the corresponding drug-containing medium and 100 *μ*L of macrophage supernatant were added to each well. After 24 h of incubation in the box, 20 *μ*L MTT (5 mg/mL) (Amresco, Solon, Ohio, USA) was added and continued to incubate for 4 h in the incubator. 150 *μ*L DMSO was added to dissolve the MTT and shaken gently for 10 mins. Absorbance was then measured at *λ* = 490 nm; the optical density (O.D.) was determined to calculate the inhibition rate. Inhibitory rate (%) = [(*C* − *T*)/*C*] × 100, where *C* is the control group and *T* is that of the treatment group.

### 2.6. Cell Cycle Analysis

H22 cells were treated with macrophages and different concentrations of GLSP (200 *μ*g/mL, 400 *μ*g/mL, and 800 *μ*g/mL) for 24 h, collected by digestion, and made into cell suspensions. The cells were washed twice with PBS (centrifuged at 1000 rpm, 5 min). The prepared single-cell suspension was stored at 4°C and washed with PBS before staining the fixing solution. Next, 100 *μ*L RNaseA was added to a 37°C water bath for 30 min and added 400 *μ*L propidium iodide (PI) staining and mixed well. Then, it was incubated at 4°C for 30 min. Flow cytometry analysis (Becton-Dickinson FACSCalibur; Becton-Dickinson, USA) detected the fluorescence of the PI-DNA complex and at 488 nm red fluorescence.

### 2.7. Annexin V-FITC/PI Double Staining Assay

H22 cells were treated with macrophages and GLSP for 24 h, and the cells were collected. Cells were washed twice with PBS. Then, 500 *μ*L of Binding Buffer was added to suspend the cells, 5 *μ*L of Annexin V-FITC was added and mixed well, and 5 *μ*L of PI was added and mixed with 5 *μ*L propidium iodide (PI) using an Annexin V-FITC/PI staining kit (KeyGEN BioTECH), Cat number: KGA105-KGA108. This was incubated for 15 min in the dark. Flow cytometry analysis was used to detect cell apoptosis.

### 2.8. Intracellular Reactive Oxygen Species (ROS) Analysis

Cellular ROS were detected using a ROS Assay Kit (KeyGEN BioTECH Co., Ltd., Nanjing, China), Cat number: KGT010-1. DCFH-DA was diluted with serum-free culture medium at 1 : 1000 to a final concentration of 10 *μ*M. After the cells were collected, they were suspended in the diluted DCFH-DA and incubated at 37°C for 20 min. The cells were mixed by inversion every 3-5 min. Cells were washed with serum-free cell culture medium three times to remove the DCFH-DA that had not entered the cells. ROS were analyzed using flow cytometry. Data processing was performed using Cell Quest.

### 2.9. Mitochondrial Membrane Potential (MMP) Analysis

Mitochondrial membrane potential (MMP) was detected using a JC-1 Apoptosis Detection Kit (KeyGEN BioTECH Co., Ltd., Nanjing, China), Cat number: KGA601-KGA604. H22 cells were treated with macrophages and GLSP for 24 h, before cell collection. 100 *μ*L 10x incubation buffer was diluted with 900 *μ*L sterile deionized water to make 1x incubation buffer. The incubation buffer was mixed and preheated to 37°C. 1 *μ*L JC-1 was added to 500 *μ*L 1x incubation buffer, vortexed, and mixed to prepare JC-1 working solution. A total of 500 *μ*L JC-1 working solution was used to suspend the cells uniformly and incubated for 15-20 min in an incubator at 37°C and 5% CO_2_. Washing was made twice with 1x incubation buffer, and 500 *μ*L of 1x incubation buffer was used to resuspend the cells.

### 2.10. Macrophage Cell Phenotype Detection

Macrophages were inoculated during the logarithmic growth phase into a six-well plate. After the drug was incubated for 24 h, the cells were collected and washed twice with PBS to collect 5 × 10^5^ cells. Then, the supernatant was removed by centrifugation, and 90 *μ*L PBS was added to resuspend the cells. The appropriate amounts of antibodies CD86 (BioLegend 105007) and CD206 (BioLegend 141703) were added. The cells were then incubated for 30 min at 37°C, 400 *μ*L PBS was added, and the cell phenotype was detected by flow cytometry.

### 2.11. Enzyme-Linked Immunosorbent Assay (ELISA) Analysis

Cytokine levels were determined using a commercial ELISA kit (Proteintech, Rosemont, IL, USA). First, all samples, reagents, and working standards were prepared as instructed by the manufacturer. The required number of microplate strips was removed, and the microwells were placed in the strip holder. Then, 100 *μ*L of each standard and sample was added to the appropriate wells. A cover seal was pressed firmly onto the top of the microwells. The plate was incubated for 90 min at 37°C in a humid environment. Then, the sealing mold was removed, 100 *μ*L was added to each well, and antibody diluent was used to dilute at 1 : 30, except for blanks. And the plates were incubated at 37°C for 1 h. 100 *μ*L of TMB was added to each well, shaken gently, and color developed at 37°C for 15 minutes. 50 *μ*L stop solution was added to each hole. Sample absorbance was read at 450 nm using a Multiskan™ GO (Thermo Fisher Scientific, Waltham, MA, USA) detector system.

### 2.12. Quantitative Real-Time PCR (RT-qPCR)

We continued to explore changes in PI3K, AKT, BAX, BCL-2, and caspase-9 mRNAs. Total RNA from each sample was extracted using a TRIzol reagent (Thermo Fisher Scientific, Waltham, MA, USA). Then, the determination of RNA concentration and purity and synthesis of cDNA first strand was carried out with 20 *μ*L system, using a RevertAid First Strand cDNA Synthesis Kit (Thermo Fisher Scientific). The sequences for primers listed in Table 1 were designed by Primer6 and then synthesized by a biotechnology company (Sangon Biotech Co., Ltd., Shanghai, China). The SYBR® Green PCR Master Mix (Thermo Fisher Scientific) was used to amplify cDNA in the Multicolor Real-time PCR Detection System (Bio-Rad Laboratories Inc.). The PCR parameters were as follows: 95°C for 5 min, followed by 40 cycles of 95°C for 15 s and 72°C for 40 s, followed by 60°C for 1 min and 95°C for 15 s. The 2^−*ΔΔ*Ct^ method was used to calculate the results.

### 2.13. Western Blot Analysis

H22 cells treated with different intervention reagents, including 800 *μ*g/mL GLSP, macrophage culture supernatant, and macrophage culture supernatant containing 800 *μ*g/mL GLSP, were added to the plates and cultured continuously for 24 h. Then, trypsin without EDTA was used for digestion, followed by centrifugation and RIPA lysis solution (Biomiga, Santiago, CA, USA) addition. In line with the molecular weight of the target protein BAX (21 kDa), BCL-2 (26 kDa), CASP-9 (46 kDa), p-AKT (60 kDa), PI3K (85 kDa), and AKT (56 kDa), proteins were transferred onto polypropylene fluoride (PVDF) membranes. Nonfat milk (5%) was used to dilute the antibody. The antibodies used were anti-BAX (50599-2-Ig, mouse polyclonal, diluted 1 : 4,000), anti-CASP-9 (10380-1-AP, rabbit polyclonal, diluted 1 : 1000), anti-PI3K (60225-1-Ig, mouse polyclonal, diluted 1 : 5000), anti-AKT (60203-2-Ig, mouse polyclonal, diluted 1 : 2000), and anti-GAPDH (60004-1-lg, mouse monoclonal, diluted 1 : 5,000). All of the above antibodies were from the Proteintech Group: anti-BCL-2 (ab182858, diluted 1 : 2,000; Abcam Group, Cambridge, MA, USA) and anti-*p*-AKT (CST 4060s, diluted 1 : 2,000; CST, MA, USA). The Tanon-5200 system (Tanon, Shanghai, China) was used for exposure. The intensity of the target protein band was read using Tanon Gis software (Tanon).

### 2.14. Statistical Analysis

Dates are presented as the mean ± standard deviation (SD). The data were analyzed using SPSS 22.0 and one-way analysis of variance (ANOVA), or a nonparametric test was used for data processing based on the normality test. And a least significant difference (LSD) method was adopted for comparisons between groups. *P* value < 0.05 was considered a statistically significant difference.

## 3. Results

### 3.1. Cytotoxic Effect of GLSP and Macrophage Supernatant on H22 Cells

**Figure 3:**
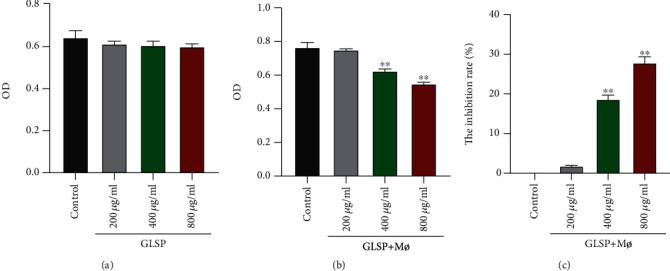
Inhibitory effects of GLSP and GLSP+M*ø* on the proliferation of H22 cells. (a) MTT assay reveals no effect on the viability of H22 cells treated with GLSP for 24 h; (b) MTT assay reveals a decrease in the viability of H22 cells treated with different concentrations of GLSP+M*ø* for 24 h, ^∗∗^*P* < 0.01 vs. control group (24 h); (c) the inhibition rate increases in H22 cells treated with different concentrations of GLSP+M*ø*. ^∗∗^*P* < 0.01 vs. H22 cell control group. GLSP: *Ganoderma lucidum* spore polysaccharide; OD: optical density.

The activity of GLSP-treated H22 cells was detected using an MTT assay. Results showed that the proliferation of H22 cells was not affected by treatment with GLSP (Figure 3(a)). However, H22 cell proliferation was notably inhibited by macrophage supernatant + 400 *μ*g/mL GLSP or +800 *μ*g/mL GLSP (*P* < 0.01) (Figure 3(b)). The results also showed a dose-dependent increase in the inhibition rate of GLSP (400 *μ*g/mL and 800 *μ*g/mL) + Mø versus the control group (*P* < 0.01) (Figure 3(c)).

### 3.2. GLSP-Activated Macrophages Induce Cell Cycle Arrest at the G2/M Phase in H22 Cells

**Figure 4:**
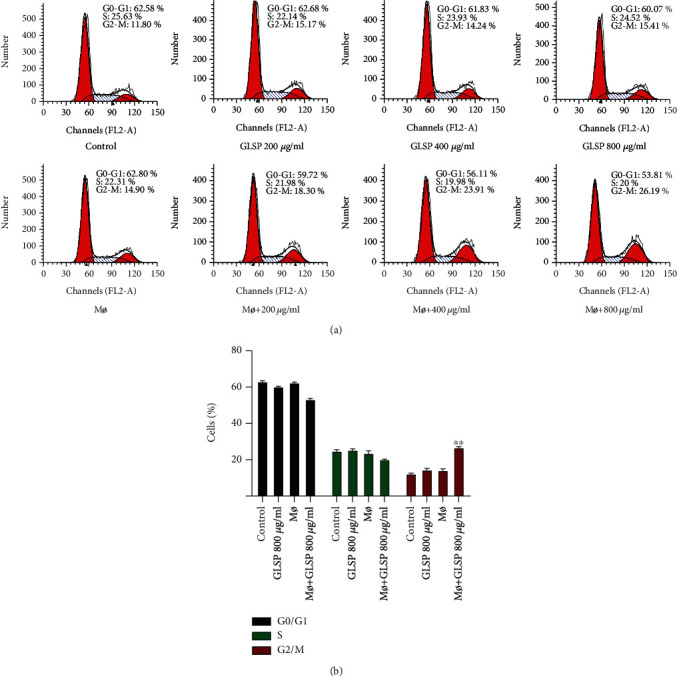
GLSP induces H22 cell arrest at the G2/M phase. (a) Cell cycle distribution of H22 cells treated with GLSP and macrophage supernatant. (b) Percentage in different periods of the cell cycle after GLSP treatment. ^∗∗^*P* < 0.01 vs. H22 cell control group. GLSP: *Ganoderma lucidum* spore polysaccharide.

We examined the cell cycle distribution after treatment with GLSP and macrophage+GLSP. The percentage of H22 cells treated with macrophages+GLSP (800 *μ*g/mL) in the G2/M phase was significantly higher than that in the control group (Figures 4(a) and 4(b)) (*P* < 0.01), whereas treatment with GLSP alone did not induce the same effect.

### 3.3. GLSP-Activated Macrophages Promote the Apoptosis of H22 Cells

**Figure 5:**
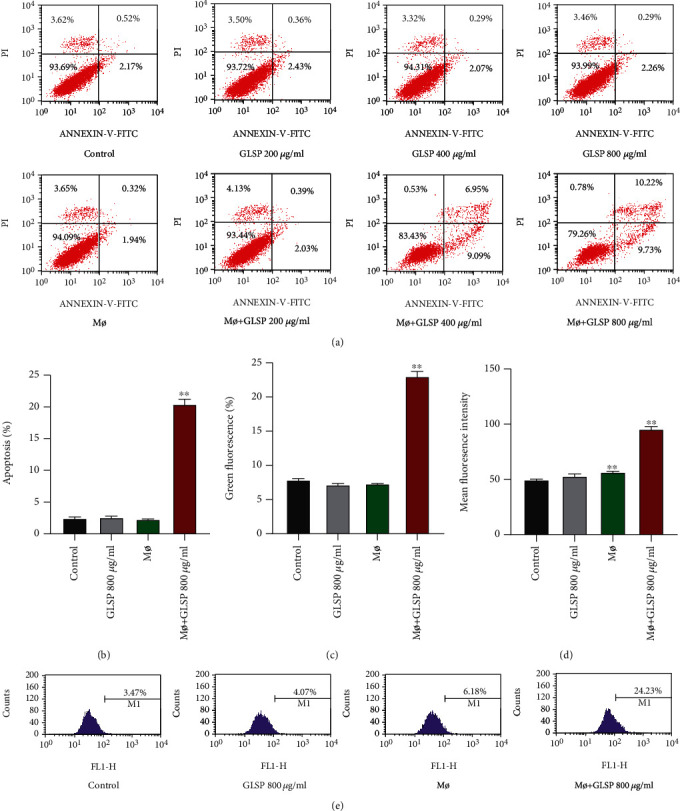
GLSP-activated macrophages induce H22 cell apoptosis. (a) Flow cytometric apoptosis in each group. (b) Percentages of apoptotic cells in each group. (c) FACS assessed MMP based on fluorescent mitochondria. (d) Analyses of ROS levels in H22 cells upon different concentrations of GLSP treatment. (e) Production of intracellular ROS in H22 detected by flow cytometry. Mean Cell Quest Pro analyzed fluorescence intensity. ^∗∗^*P* < 0.01 vs. the control group. GLSP: *Ganoderma lucidum* spore polysaccharide; ROS: reactive oxygen species.

Results showed that the percentage of apoptotic H22 cells was significantly increased upon macrophage+GLSP treatment (Figures 5(a) and 5(b)) (*P* < 0.01). These results indicated that GLSP-activated macrophages effectively induced H22 cell apoptosis.

We also examined GLSP-induced changes in MMP. GLSP-activated macrophage treatment induced the conversion of red fluorescence to green fluorescence, indicating a decrease in MMP (Figure 5(c)) (*P* < 0.01). As ROS generation is closely related to mitochondrial apoptosis, ROS was detected to test whether oxidative stress had an effect on GLSP-activated macrophage-induced apoptosis in H22 cells. As shown in Figures 5(d) and 5(e), GLSP-activated macrophages led to a significant increase in intracellular ROS levels compared to that of the control group (*P* < 0.01). Therefore, the elevation of ROS production may be a relevant cause of GLSP-activated macrophage-induced apoptosis.

### 3.4. GLSP Activate Macrophage Polarization

**Figure 6:**
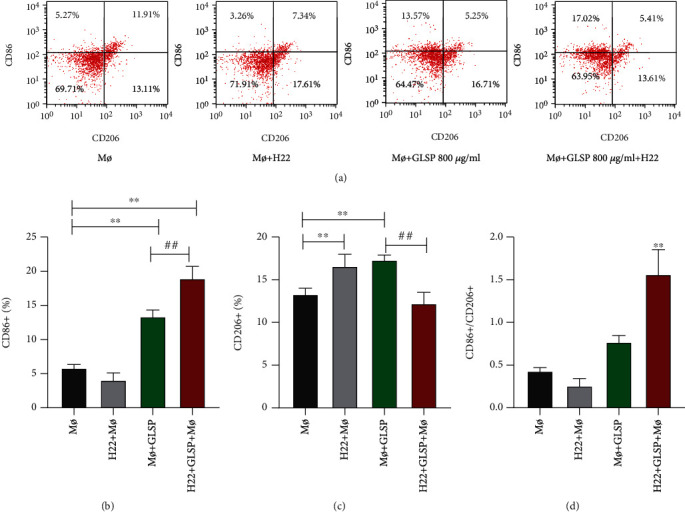
Effect of GLSP on the expression of M1 and M2 macrophage markers: (a) triple staining of flow cytometry analysis of macrophage cells; (b) CD86+ macrophage cells; (c) CD206+ macrophage cells; (d) CD86+/CD206+ macrophage cells. ^∗∗^*P* < 0.01 vs. the control group, ^##^*P* < 0.01 vs. the M*ø*+GLSP group. GLSP: *Ganoderma lucidum* spore polysaccharide.

Macrophages are dynamic cells that react to different stimuli by adjusting their functional state. Classically activated macrophages, M1 type, and alternatively activated M2 type are the two extremes of this state. We investigated the polarizing effect of GLSP treatment on macrophages. CD86 analysis revealed that H22 tumor cells had no effect on macrophage polarization, but GLSP treatment could activate macrophages, which polarized towards M1 type (*P* < 0.01) (Figures 6(a) and 6(b)). CD206 analysis revealed that H22 tumor cells and GLSP independently could increase M2 type macrophages (*P* < 0.01), but H22 GLSP+macrophages reduced the amount of M2 type (Figures 6(a) and 6(c)). As shown in Figure 5(d), H22+macrophages+GLSP improved the ratio of M1/M2. Therefore, GLSP increases the expression of M1 type macrophages and decreases the expression of M2 type macrophages. The cocultivation group improved the ratio of M1/M2, which affects the polarization of macrophages.

### 3.5. Effect of GLSP on Cytokine Production

**Figure 7:**
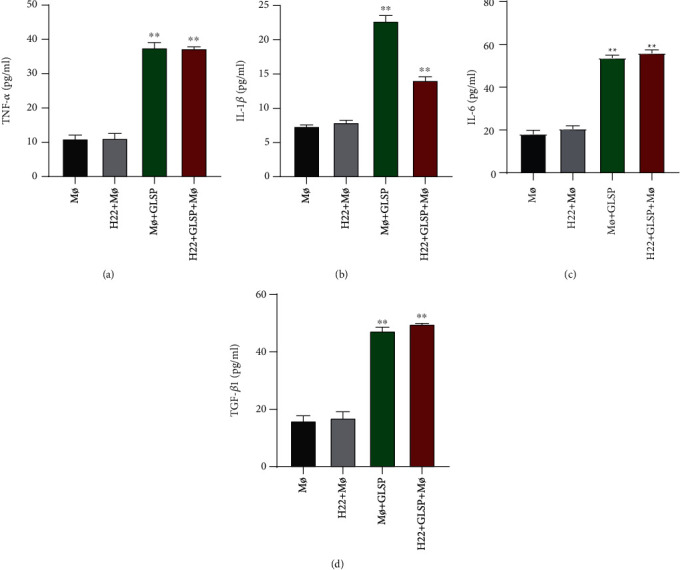
Increasing of proinflammatory cytokine expression by GLSP in macrophages. (a) TNF-*α* levels; (b) IL-1*β* levels; (c) IL-6 levels; (d) TGF-*β*1 levels. Data is expressed as the mean ± SD (*n* = 5), ^∗∗^*P* < 0.01 vs. the control group; ^##^*P* < 0.01 vs. the H22+macrophage group. GLSP: *Ganoderma lucidum* spore polysaccharide.

As shown in Figure 7, the TNF-*α* (Figure 7(a)), IL-1*β* (Figure 7(b)), IL-6 (Figure 7(c)), and TGF-*β*1 (Figure 7(d)) levels were significantly higher in the macrophage+GLSP group than in the control group (*P* < 0.01) and in the H22+macrophage+GLSP group than in the H22+macrophage group (*P* < 0.01).

### 3.6. GLSP Activate Macrophages Affecting the PI3K/AKT and Mitochondria-Mediated Apoptotic Signaling Pathways

**Figure 8:**
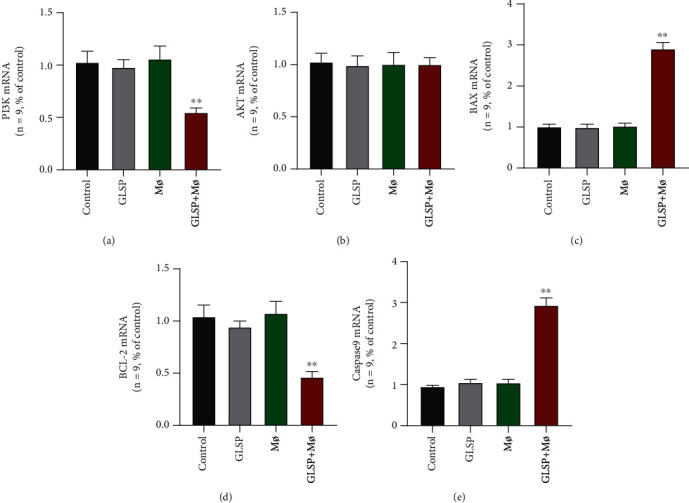
Effect of GLSP and M*ø* on the mRNA levels of PI3K/AKT and mitochondria-mediated apoptotic signaling pathway genes. H22 was treated with GLSP or M*ø* for 24 h, and then, the (a) mRNA levels of PI3K, (b) AKT, (c) BAX, (d) BCL-2, and (e) CASP-9 were detected by qPCR. The histogram bars represent three independent experiments, and the values are the mean ± SD. ^∗∗^*P* value < 0.01 versus the control group. GLSP: *Ganoderma lucidum* spore polysaccharide.

In the above experiments, GLSP activate macrophages effectively, thereby inducing apoptosis and other changes in H22 cells. Thus, we used RT-qPCR and western blot to explore the differences in apoptotic cell molecules and the PI3K/AKT signaling pathway. At the genetic level, the levels of PI3K were significantly decreased in GLSP+macrophage-treated H22 cells compared with the control group (*P* < 0.01) (Figure 8(a)). In Figure 8(b), the levels of AKT were not affected in GLSP+macrophage-treated H22 cells versus the control group. We measured the levels of BAX, BCL-2, and CASP-9. The levels of proapoptotic BAX were markedly increased, and antiapoptotic BCL-2 was significantly decreased in GLSP+macrophage-treated H22 cells versus the control group (*P* < 0.01) (Figures 8(c) and 8(d)). Levels of CASP-9 were increased in GLSP+macrophage-treated H22 cells versus control cells (*P* < 0.01) (Figure 8(e)).

**Figure 9:**
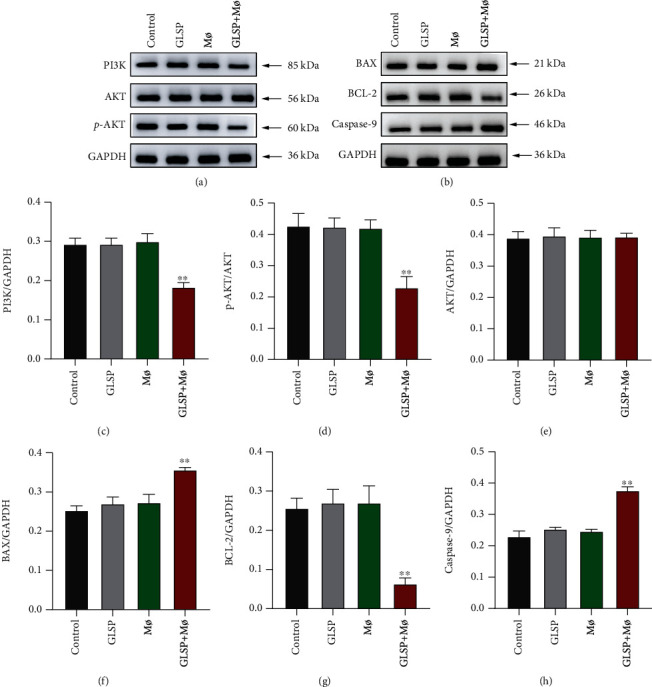
Effect of GLSP and M*ø* on the expression of PI3K/AKT and mitochondria-mediated apoptotic signaling pathway proteins. H22 cells were treated with GLSP or M*ø* for 24 h, and then, the protein levels of PI3K, AKT, p-AKT, BAX, BCL-2, and CASP-9 were detected by WB. (a) Protein expression of PI3K/AKT signal pathway; (b) protein expression of BAX, BCL-2, and CASP-9; (c) relative PI3K/GAPDH protein; (d) relative p-AKT/AKT protein; (e) relative AKT/GAPDH protein; (f) relative BAX/GAPDH protein; (g) relative BCL-2/GAPDH protein; (h) relative caspase-9/GAPDH protein, and values are the mean ± SD. ^∗∗^*P* value < 0.01 vs. control group. GLSP: *Ganoderma lucidum* spore polysaccharide.

We continued to explore changes at the protein level. In Figures 8(a) and 8(c), the levels of PI3K were significantly decreased in GLSP+macrophage-treated H22 cells versus the control group (*P* < 0.01). Similarly, the levels of p-AKT were significantly decreased in GLSP+macrophage-treated H22 cells (*P* < 0.01) (Figures 9(a) and 9(d)). In Figure 8(e), the levels of AKT were not affected in GLSP+macrophage-treated H22 cells. We also measured the protein levels of BAX, BCL-2, and CASP-9 and found that the levels of proapoptotic BAX were markedly increased (*P* < 0.01) (Figures 9(b) and 9(f)), and the level of antiapoptotic BCL-2 was significantly decreased in GLSP+macrophage-treated H22 cells versus control groups (*P* < 0.01) (Figures 9(b) and 9(g)). Levels of CASP-9 were increased in GLSP+macrophage-treated H22 cells versus control cells (*P* < 0.01) (Figure 9(h)). These results indicated that GLSP+macrophages could simultaneously activate genes and proteins in the mitochondria-mediated apoptotic signaling pathway.

## 4. Discussion

The primary characteristics of tumors are malignant proliferation and imbalance between cell proliferation and apoptosis [23]. Inhibiting proliferation and inducing apoptosis are excellent strategies for tumor treatment [24]. Previous research has shown that GLSP and *G. lucidum* triterpenes in *G. lucidum* spore powder can effectively inhibit tumors [25, 26]. The antitumor mechanism of *G. lucidum* spore powder includes inhibition of tumor cell proliferation, induction of tumor cell apoptosis, and termination of the tumor cell cycle. Early experiments in our group had found that the water extract of *G. lucidum* spores showed no apparent inhibitory effect on tumor cells and no cytotoxicity change. However, after coculture with immune cells such as macrophages, it showed inhibition of tumor cell characteristics. In order to determine the mechanism of action of the antitumor effect of *G. lucidum* spores, we separately studied GLSP, *G. lucidum* spore triterpene, and *G. lucidum* spore oil. Finally, it was discovered that GLSP is the material basis for activating macrophages to enhance immunity and antitumor activity.

GLSP can activate the immune response. It improved the immune state to achieve the balance of the body's immune state and inhibit the development of tumors [27]. MTT results showed that GLSP alone did not affect the proliferation of H22 tumor cells, showing no cytotoxicity. Nevertheless, it inhibited H22 tumor cells by activating macrophages. In our cell cycle experiments, we found that the macrophage supernatant containing GLSP can block tumor cells in the G2/M stage, whereas the macrophage supernatant alone had no blocking effect on H22 tumor cells. Cell cycle arrest at the G2/M phase shows that there is damage in the intracellular DNA, which is challenging to repair.

**Figure 10:**
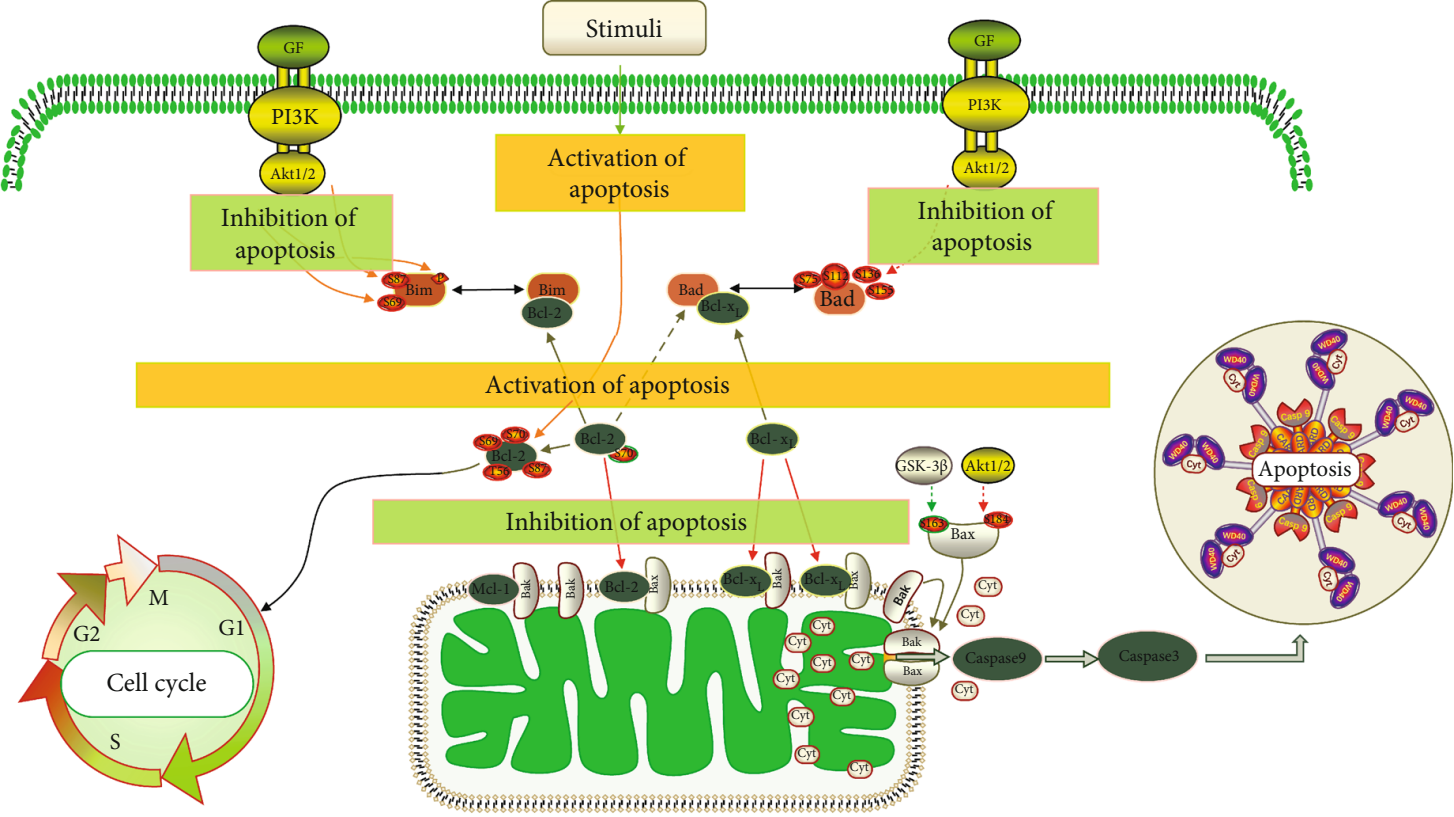
Schematic representation of the endogenous mitochondrial apoptosis pathway.

The mitochondrial apoptosis pathway is an integral part of the endogenous cell apoptosis regulation [28]. The endogenous pathway is activated by cellular stress, DNA damage, developmental signals, and loss of survival factors [29]. BCL-2 family proteins are composed of proapoptotic factors (BAX, Bad, Bak, and Noxa) and antiapoptotic factors (BCL-2, BCL-xL, BCL-w, and Mel-1) [30]. The BCL-2 family members are located on the mitochondria and can control its permeability, the release of cytochrome C, the activation of “priming” CASP-9, and the subsequent activation of “executive” CASP-3. Endogenous apoptosis can be inhibited through prosurvival signaling pathways such as PI3K/AKT and MAPK [31, 32]. The PI3K/AKT pathway is an intracellular signaling pathway with phosphatidylinositol kinase and serine/threonine kinase activity. It is involved in regulating cell proliferation, apoptosis, survival, growth, and other cellular physiological functions, and these processes are known to be affected in tumors [33]. The regulation of PI3K/AKT activation is one of the hot topics in tumor pharmacology. As downstream molecules of PI3K/AKT, BCL-2 family proteins play a vital role in regulating apoptosis, mainly through endogenous pathways. Many survival factors can activate the PI3K pathway, leading to the activation of AKT, and AKT plays an essential role in cell survival signal transduction. PTEN has a negative regulatory effect on the PI3K/AKT pathway [34]. Activated AKT can phosphorylate and inhibit Bad, BAX, CASP-9, GSK-3, and FOXO1 [32]. Western blot and RT-qPCR results showed that GLSP downregulated the expression of PI3K and p-AKT genes and proteins in H22 cells. Our results showed a reduced phosphorylation level of AKT by inhibition of the PI3K/AKT signaling pathway, which simultaneously downregulates the expression of BCL-2 in H22 cells at mRNA and posttranslational levels and upregulates the expression of BAX, indicating that GLSP can inhibit the PI3K/AKT signaling pathway and induce apoptosis in liver cancer H22 cells (Figure 10).

The monocyte-macrophage system is an essential part of innate immunity [35, 36]. During inflammation or infection, monocytes in the blood are recruited into the tissue and differentiate into mature macrophages, a group of highly heterogeneous cells. Depending on the microenvironment, macrophages can polarize into different functional phenotypes [37]. According to their different activation states, they are mainly divided into classically activated macrophages (M1 type) and alternatively activated macrophages (M2 type) [38]. The polarization of phagocytic cells is affected by various cytokines in the microenvironment [39]. When the epithelial barrier is destroyed and pathogenic microorganisms invade, a large number of circulating monocytes are recruited under the action of chemokines and differentiate into proinflammatory cells, namely, M1 macrophages, induced by local cytokines [40]. M1 macrophages have potent cytotoxicity; are highly sensitive to LPS; secrete many inflammatory factors and reactive oxygen products, such as IL-6, IL23, and TNF-*α*; promote inflammation cascades and tissue damage; activate Th1/Th17 adaptive immunity; and promote the elimination of pathogenic microorganisms. M2 type macrophages also increase during the disease, inhibit the inflammatory response, avoid excessive damage to the tissue, and, at the same time, remove pathogenic bacteria and cell debris in the process of inflammation subsiding. They also promote tissue repair and immune balance. The immune balance of the intestine depends on the two types of macrophages working together and coordinating with each other. Therefore, regulating the balance between M1 and M2 macrophages is essential for the occurrence and development of cancer [41].

TAMs are derived from monocytes in the blood system and enter tumor tissues under the action of chemokines [42]. The colony-stimulating factor secreted by tumor cells can prolong the survival time of TAMs. When TAMs are moderately activated in the tumor environment (M1 type), they exert antitumor immune function, which can kill tumor cells and destroy vascular endothelium, thereby inhibiting tumor development. However, if this stimulus is not suppressed in a short time, TAMs will be polarized into M2 type under the action of various cytokines secreted by tumor cells, which is why most TAMs in tumor tissues are M2 type [38]. In contrast to the M1 type, M2 TAMs can secrete growth factors, angiogenesis factors, and proteases, thereby stimulating tumor cell proliferation, promoting angiogenesis and tumor cell invasion and migration, and escaping the surveillance of antitumor immunity [43]. Therefore, the induction of secondary polarization of TAMs in tumor tissues and the transformation of M2 TAMs to M1 have become an essential target for tumor therapy in recent years [44]. Previous research has found that glycopeptide derived from G. lucidum (Gl-PS) could promote polarization of M1 macrophage vs. M2 macrophages [45]. In our macrophage typing experiments, GLSP can increase the number of CD86+ cells, which is an M1 macrophage marker. When H22 tumor cells were cocultured with macrophages, we found that GLSP decreased the number of CD206+ macrophages, an M2 type marker. Overall, when H22 tumor cells are cocultured with macrophages in the TME, GLSP increases the ratio of M1/M2 macrophages. Therefore, we can speculate that GLSP has a regulatory effect on M1 and M2 macrophages in the tumor microenvironment.

Currently, chemotherapy is one of the most common cancer treatments, but it has noticeable side effects [46]. In contrast, “nutritional drugs” are known for their low toxicity. “Nutrition” is a concept that has attracted much attention to prevent and treat diseases [47]. Traditional Chinese medicine (TCM) is a rich source of nutritional medicine that has been used for thousands of years [48]. It has an excellent effect on treating many chronic diseases. Additionally, TCM can also be used as part of a daily diet. TCM is a safe and effective way to prevent and treat diseases. GLSP is one of the foremost effective ingredients in TCM as it has a wide range of therapeutic effects and relatively low toxicity. It is a promising nutritional drug and has attracted wide attention in biomedicine in recent years [49, 50]. The above studies revealed that GLSP activates macrophages to induce apoptosis of H22 hepatocellular carcinoma cell in vitro and the biological mechanism. Next, we will continue to verify the biological activity of GLSP to enhance immunity and antitumor in vivo.

## 5. Conclusions

In summary, GLSP reshapes the tumor microenvironment by activating macrophages, regulating the polarization of macrophages, and promoting the secretion of various inflammatory factors and cytokines. Moreover, we found that GLSP can block H22 tumor cells in the G2/M phase by activating macrophages and can activate PI3K/AKT signaling pathways to affect the mitochondrial apoptotic pathway and promote tumor cell apoptosis. Therefore, as a natural nutrient, GLSP can alter macrophage polarity and has potential to reshape the tumor microenvironment activity.

## Figures and Tables

**Table 1:** Primer sequences used in the RT-qPCR analysis.

Gene		Sequences (5′-3′)
PI3K	Forward	AGGGAAGCGAGACGGCACTTT
	Reverse	CCACTACGGAGCAGGCATAGCA
AKT	Forward	CCAAGCACCGTGTGACCATGAA
	Reverse	TGGCGACGATGACCTCCTTCTT
BAX	Forward	CCAGGATGCGTCCACCAAGAAG
	Reverse	CCGTGTCCACGTCAGCAATCAT
BCL-2	Forward	TGCCACCTGTGGTCCATCTGA
	Reverse	CTCTGCGAAGTCACGACGGTAG
Caspase-9	Forward	GCCACTGCCTCATCATCAACAA
	Reverse	AGCGGAATCGGTGCTCAAGTT

## Data Availability

The data (original) used to support the findings of this study are available from the authors upon request.

## References

[1] Jin H., Wang S., Zaal E. A. (2020). A powerful drug combination strategy targeting glutamine addiction for the treatment of human liver cancer. *eLife*.

[2] Degroote H., Van Dierendonck A., Geerts A., Van Vlierberghe H., Devisscher L. (2018). Preclinical and clinical therapeutic strategies affecting tumor-associated macrophages in hepatocellular carcinoma. *Journal of Immunology Research*.

[3] Lee H. S., Oh D. S. (2020). Assessing the anti-cancer therapeutic mechanism of a herbal combination for breast cancer on system-level by a network pharmacological approach. *Anticancer Research*.

[4] Han L., Cao X., Chen Z. (2020). Overcoming cisplatin resistance by targeting the MTDH-PTEN interaction in ovarian cancer with sera derived from rats exposed to Guizhi Fuling wan extract. *BMC complementary medicine and therapies*.

[5] Zhao B. B., Ye Z. H., Gao X., Li H. M. (2019). Diwu Yanggan modulates the Wnt/*β*-catenin pathway and inhibits liver carcinogenesis signaling in 2-AAF/PH model rats. *Current medical science*.

[6] Lau M. F., Chua K. H., Sabaratnam V., Kuppusamy U. R. (2020). In vitroandin silicoanticancer evaluation of a medicinal mushroom,Ganoderma neo‐japonicumImazeki, against human colonic carcinoma cells. *Biotechnology and Applied Biochemistry*.

[7] Wang G., Zhang J., Mizuno T. (1993). Antitumor active polysaccharides from the Chinese MushroomSongshan lingzhi, the fruiting body ofGanoderma tsugae. *Bioscience, Biotechnology, and Biochemistry*.

[8] Li D., Zhong Q., Liu T., Wang J. (2016). Cell growth stimulating effect of Ganoderma lucidum spores and their potential application for Chinese hamster ovary K1 cell cultivation. *Bioprocess and Biosystems Engineering*.

[9] Li Z., Shi Y., Zhang X. (2020). Screening immunoactive compounds of *Ganoderma lucidum* spores by mass spectrometry molecular networking combined with *in vivo* zebrafish assays. *Frontiers in Pharmacology*.

[10] Tsutsumi S., Tokunaga Y., Shimizu S. (2020). Effects of indole and indoxyl on the intracellular oxidation level and phagocytic activity of differentiated HL-60 human macrophage cells. *The Journal of Toxicological Sciences*.

[11] Graziano F., Vicenzi E., Poli G. (2016). Plastic restriction of HIV-1 replication in human macrophages derived from M1/M2 polarized monocytes. *Journal of Leukocyte Biology*.

[12] Zhou J., Zhang A., Fan L. (2020). HSPA12B secreted by tumor-associated endothelial cells might induce M2 polarization of macrophages via activating PI3K/Akt/mTOR signaling. *Oncotargets and Therapy*.

[13] le Y., Cao W., Zhou L. (2020). Infection of *Mycobacterium tuberculosis* promotes both M1/M2 polarization and MMP production in cigarette smoke-exposed macrophages. *Frontiers in Immunology*.

[14] Zhang F., Xuan Y., Cui J., Liu X., Shao Z., Yu B. (2017). Nicorandil modulated macrophages activation and polarization via NF-*κ*b signaling pathway. *Molecular Immunology*.

[15] Ma D., Zhang Y., Chen G., Yan J. (2020). miR-148a affects polarization of THP-1-derived macrophages and reduces recruitment of tumor-associated macrophages via targeting SIRP*α*. *Cancer Management and Research*.

[16] Kozak J., Forma A., Czeczelewski M. (2020). Inhibition or reversal of the epithelial-mesenchymal transition in gastric cancer: pharmacological approaches. *International journal of molecular sciences*.

[17] Lan H., Zhang W., Jin K., Liu Y., Wang Z. (2020). Modulating barriers of tumor microenvironment through nanocarrier systems for improved cancer immunotherapy: a review of current status and future perspective. *Drug Delivery*.

[18] Meng Q., Luo X., Chen J. (2020). Unmasking carcinoma-associated fibroblasts: key transformation player within the tumor microenvironment. *Biochimica Et Biophysica Acta. Reviews on Cancer*.

[19] Eyre J. J., Williams R. L., Levis H. J. (2020). A human retinal microvascular endothelial-pericyte co-culture model to study diabetic retinopathy in vitro. *Experimental Eye Research*.

[20] Yeh C. H., Chen H. C., Yang J. J., Chuang W. I., Sheu F. (2010). Polysaccharides PS-G and protein LZ-8 from Reishi (Ganoderma lucidum) exhibit diverse functions in regulating murine macrophages and T lymphocytes. *Journal of Agricultural and Food Chemistry*.

[21] Wang Y., Liu Y., Yu H. (2017). Structural characterization and immuno-enhancing activity of a highly branched water-soluble *β*-glucan from the spores of _Ganoderma lucidum_. *Carbohydrate Polymers*.

[22] Chen Y., Lv J., Li K. (2016). Sporoderm-broken spores of Ganoderma lucidum inhibit the growth of lung cancer: involvement of the Akt/mTOR signaling pathway. *Nutrition and Cancer*.

[23] Gao L., Jin H. J., Zhang D., Lin Q. (2020). Silencing circRNA_001937 may inhibit cutaneous squamous cell carcinoma proliferation and induce apoptosis by preventing the sponging of the miRNA-597-3p/FOSL2 pathway. *International Journal of Molecular Medicine*.

[24] Sun Y., Liu Y., Cai Y. (2020). Downregulation of LINC00958 inhibits proliferation, invasion and migration, and promotes apoptosis of colorectal cancer cells by targeting miR-3619-5p. *Oncology Reports*.

[25] Zhang S., Nie S., Huang D., Huang J., Feng Y., Xie M. (2014). *Ganoderma atrum* polysaccharide evokes antitumor activity *via* cAMP-PKA mediated apoptotic pathway and down-regulation of Ca^2+^/PKC signal pathway. *Food and Chemical Toxicology*.

[26] Zhang S., Nie S., Huang D., Huang J., Wang Y., Xie M. (2013). Polysaccharide from Ganoderma atrum evokes antitumor activity via toll-like receptor 4-mediated NF-*κ*B and mitogen-activated protein kinase signaling pathways. *Journal of Agricultural and Food Chemistry*.

[27] Lin K. I., Kao Y. Y., Kuo H. K. (2006). Reishi polysaccharides induce immunoglobulin production through the TLR4/TLR2-mediated induction of transcription factor Blimp-1. *The Journal of Biological Chemistry*.

[28] Xu Y., Zheng J., Sun P. (2020). Cepharanthine and curcumin inhibited mitochondrial apoptosis induced by PCV2. *BMC Veterinary Research*.

[29] Wang X., Liu Q., Kong D. (2020). Down-regulation of SETD6 protects podocyte against high glucose and palmitic acid-induced apoptosis, and mitochondrial dysfunction via activating Nrf2-Keap1 signaling pathway in diabetic nephropathy. *Journal of Molecular Histology*.

[30] Godwin C. D., Bates O. M., Jean S. R. (2020). Anti-apoptotic BCL-2 family proteins confer resistance to calicheamicin-based antibody-drug conjugate therapy of acute leukemia. *Leukemia & Lymphoma*.

[31] Dong B., Yang Z., Ju Q. (2020). Anticancer effects of Fufang Yiliu Yin formula on colorectal cancer through modulation of the PI3K/Akt pathway and BCL-2 family proteins. *Frontiers in Cell and Development Biology*.

[32] Robert A., Pujals A., Favre L., Debernardi J., Wiels J. (2020). The BCL-2 family protein inhibitor ABT-737 as an additional tool for the treatment of EBV-associated post-transplant lymphoproliferative disorders. *Molecular Oncology*.

[33] Ma K., Zhang C., Li W. (2020). Gamabufotalin suppressed osteosarcoma stem cells through the TGF-*β*/periostin/PI3K/AKT pathway. *Chemico-Biological Interactions*.

[34] Zhang S., Cui R. (2020). The targeted regulation of miR-26a on PTEN-PI3K/AKT signaling pathway in myocardial fibrosis after myocardial infarction. *European Review for Medical and Pharmacological Sciences*.

[35] Al-Sarireh B., Eremin O. (2000). Tumour-associated macrophages (TAMS): disordered function, immune suppression and progressive tumour growth. *Journal of the Royal College of Surgeons of Edinburgh*.

[36] Yang W., Lu Y., Xu Y. (2012). Estrogen represses hepatocellular carcinoma (HCC) growth via inhibiting alternative activation of tumor-associated macrophages (TAMs). *The Journal of Biological Chemistry*.

[37] Shen Z., Seppänen H., Vainionpää S. (2012). IL10, IL11, IL18 are differently expressed in CD14^+^ TAMs and play different role in regulating the invasion of gastric cancer cells under hypoxia. *Cytokine*.

[38] Almatroodi S. A., McDonald C. F., Darby I. A., Pouniotis D. S. (2016). Characterization of M1/M2 tumour-associated macrophages (TAMs) and Th1/Th2 cytokine profiles in patients with NSCLC. *Cancer Microenvironment*.

[39] Komohara Y., Takeya M. (2017). CAFs and TAMs: maestros of the tumour microenvironment. *The Journal of Pathology*.

[40] Bi S., Huang W., Chen S. (2020). *Cordyceps militaris* polysaccharide converts immunosuppressive macrophages into M1-like phenotype and activates T lymphocytes by inhibiting the PD-L1/PD-1 axis between TAMs and T lymphocytes. *International Journal of Biological Macromolecules*.

[41] Yin M., Shen J., Yu S. (2019). Tumor-associated macrophages (TAMs): a critical activator in ovarian cancer metastasis. *Oncotargets and Therapy*.

[42] Li Y., Cao F., Li M. (2018). Hydroxychloroquine induced lung cancer suppression by enhancing chemo-sensitization and promoting the transition of M2-TAMs to M1-like macrophages. *Journal of Experimental & Clinical Cancer Research*.

[43] Soave D. F., Miguel M. P., Tomé F. D., de Menezes L. B., Nagib P. R., Celes M. R. (2016). The fate of the tumor in the hands of microenvironment: role of TAMs and mTOR pathway. *Mediators of Inflammation*.

[44] Wang X., Jiao X., Meng Y. (2018). Methionine enkephalin (MENK) inhibits human gastric cancer through regulating tumor associated macrophages (TAMs) and PI3K/AKT/mTOR signaling pathway inside cancer cells. *International Immunopharmacology*.

[45] Sun L. X., Lin Z. B., Lu J. (2017). The improvement of M1 polarization in macrophages by glycopeptide derived from Ganoderma lucidum. *Immunologic Research*.

[46] Xu Q., Yan Y., Gu S. (2018). A novel inflammation-based prognostic score: the fibrinogen/albumin ratio predicts prognoses of patients after curative resection for hepatocellular carcinoma. *Journal of Immunology Research*.

[47] Richards D. J. (1993). Nutritional products as drugs or food implications for development. *Aging (Milano)*.

[48] Hua S., Zhang Y., Liu J. (2018). Ethnomedicine, phytochemistry and pharmacology ofSmilaxglabra: an important traditional Chinese medicine. *The American Journal of Chinese Medicine*.

[49] Chen S. D., Hsieh M. C., Chiou M. T., Lai Y. S., Cheng Y. H. (2008). Effects of fermentation products ofGanoderma lucidumon growth performance and immunocompetence in weanling pigs. *Archives of Animal Nutrition*.

[50] Chiu S. W., Wang Z. M., Leung T. M., Moore D. (2000). Nutritional value of _Ganoderma_ extract and assessment of its genotoxicity and anti-genotoxicity using comet assays of mouse lymphocytes. *Food and chemical toxicology*.

